# Effect of Thixotropic Agent on the Color Stability of Platinum-Based Silicone Maxillofacial Elastomers after Artificial Aging

**DOI:** 10.3390/ma16175867

**Published:** 2023-08-27

**Authors:** Sudarat Kiat-amnuay, Pinar Cevik, Cem Kurtoglu

**Affiliations:** 1 Department of General Practice and Dental Public Health, School of Dentistry, The University of Texas Health Science Center at Houston, Houston, TX 77054, USA; 2 Houston Center for Biomaterials and Biomimetics, Houston, TX 77054, USA; 3 Department of Prosthodontics, Faculty of Dentistry, Gazi University, Ankara 06490, Türkiye; pinarcevik@gazi.edu.tr; 4 Department of Prosthodontics, Faculty of Dentistry, Cukurova University, Adana 01250, Türkiye

**Keywords:** aging, color, maxillofacial prosthesis, prosthodontics, silicones, thixotropic agent, viscosity

## Abstract

Maxillofacial prostheses are essential for restoring natural appearance and function in individuals with defects in the head and neck regions. Thixotropic agents, as liquid additives, are known to increase the viscosity of silicone elastomers. However, color deterioration remains a challenge in facial prostheses, leading to the need for refabrication. Despite this, there is limited research on the effect of thixotropic agents on the color stability of silicone maxillofacial elastomers. This study aims to investigate the impact of different thixotropic agent amounts on the color degradation of various maxillofacial silicone elastomers. Three elastomers (A-2000, A-2006, and A-2186) were combined with five pigments (no pigment as control, red, yellow, blue, and a mixture of red, yellow, and blue), and mixed with six thixotropic agent quantities (0, 1, 2, 3, 4, and 5 drops). A total of 450 specimens were fabricated (n = 5) and aged in an artificial aging chamber. L*, a*, b* readings were obtained before and after aging using a digital spectrophotometer. Color difference (ΔE*) means and standard deviations for 150 kj/m^2^, 300 kj/m^2^, and 450 kj/m^2^ were calculated. Statistical analyses, including four-way ANOVA and Fisher’s PLSD test, were conducted to determine any significant differences (*p* < 0.05) among the groups. A comprehensive analysis revealed significant four-way interactions among the groups. In the mixed-pigmentation group, adding 4 drops of thixotropic agent resulted in ΔE* above 3 only in A-2186 silicone at 300 and 450 kj/m^2^ energy levels. However, the color stability of mixed-pigmented A-2000 and A-2006 remained within the acceptable thresholds of 3 ΔE* at all irradiance levels in this study. At each energy level, A-2006 exhibited the highest color stability with an increasing thixotropic agent quantity among all the silicones. Conversely, A-2186 was more affected by the increased number of thixotropic agent drops in each pigmentation group, including the control group at 450 kj/m^2^. The quantity of thixotropic agent plays a crucial role in determining the color stability of different silicone elastomers pigmented with various intrinsic pigments. The thixotropic agent amount has a more significant impact on color stability than the type of pigment used in the silicone elastomers. A key overarching insight from this investigation is the identification of a safety threshold for the thixotropic agent quantity of 3 drops for each silicone type, pigmentation, and energy level. These findings highlight the importance of considering the proper combination of thixotropic agents, pigments, and silicone materials to achieve optimal color stability in maxillofacial prosthetic applications.

## 1. Introduction

Maxillofacial prostheses play a crucial role in restoring natural and functional appearances for individuals with congenital, developmental, or acquired defects in the head and neck regions [1,2,3,4]. The success of such prosthetics depends on various factors, including the size and nature of the defect, the expertise of the maxillofacial prosthodontist or anaplastologist, and the properties of the materials employed [5,6,7]. Silicone elastomers have emerged as the preferred choice for maxillofacial prosthetics. The unique properties of silicone elastomers make them well-suited for this application, allowing for realistic and comfortable restorations that blend seamlessly with the surrounding tissues. As a result, they have become a widely used material in the field of maxillofacial prosthetics [8,9,10].

In recent years, significant advancements have been made in enhancing the mechanical and physical properties of silicone elastomers used in facial prostheses [11,12]. However, despite these improvements, color instability remains a critical limitation, leading to relatively short lifespans for these prostheses, typically ranging from 3 months to 1 year [2,11]. The primary factors contributing to color instability are exposure to ultraviolet (UV) light, air pollution, cosmetics, and the use of strong solvents during the cleaning process [13,14,15,16]. These external factors can cause gradual changes in the prosthesis’s color, affecting its aesthetic appeal and overall lifespan. As a result, further research is essential to address these challenges and extend the longevity of facial prostheses.

The CIE Lab* system is a color space defined by the International Commission on Illumination (abbreviated CIE) in 1976 [17]. The CIELAB system is a frequently used color notation system that is based on the three basic dimensions of color characterization: hue (color itself), value (lightness, achromatic, from black to white), and chroma (color strength, from pale to strong) [18]. It is used for the color stability of materials subjected to artificial aging or natural weathering [19,20,21,22,23,24]. A crucial aspect is measuring the color change (ΔE*) of materials to determine their color degradation over time. In the field of maxillofacial prosthodontics, it is essential to consider the thresholds for visual perceptibility and acceptability when reporting color changes. The acceptability threshold is defined as ΔE* = 3.0, while the perceptibility threshold is ΔE* = 1.1 [25]. Therefore, it is important to evaluate the color results by considering the acceptable and perceptible thresholds.

Artificial aging may not fully reflect natural weathering. On the other hand, artificial aging presents a perfect concept of implementing the effect of different outdoor conditions on the properties of materials. In this regard, the usage of lighting energy in kj/m^2^ is recommended instead of reporting the total hours of aging in the artificial aging process [22]. Nevertheless, this method can be useful for comparing the color stability of different dental materials.

Thixotropic agents, also known as viscosity modifiers, have been recognized as a potential solution to improve the handling of silicone elastomers, especially for facial silicone elastomers that have low viscosity. They are also used as anti-air bubbling agent when vacuum mixing is not available [26,27]. Usually, a recommended dosage of one to two thixotropic agent drops is suggested for numerous platinum silicone elastomers, according to the manufacturers [26]. However, due to the variations in viscosity among silicone products, the effects of thixotropic agents may differ depending on the specific silicone material.

There is no existing information on the effect of thixotropic agents at present on the color stability of maxillofacial silicone elastomers. Consequently, the purpose of this study is to assess the influence of different drops of thixotropic agent on the color stability of three platinum silicone elastomers after being aged in an artificial aging chamber at different energy levels. The null hypothesis was that the addition of the thixotropic agent would have no effect on the color stability of pigmented silicone after undergoing artificial aging.

## 2. Materials and Methods

A total of 450 silicone specimens were fabricated from three commonly used silicone elastomers (A-2000, A-2006, and A-2186; Factor II Inc., Lakeside, AZ, USA) combined with five different pigments (no pigment (control), red (R), yellow (Y), blue (B), and a mixture of R+Y+B; Factor II Inc., Lakeside, AZ, USA) and various thixotropic agent quantities (0, 1, 2, 3, 4, and 5 drops) (Thixo, Factor II Inc., Lakeside, AZ, USA). Five specimens for each experimental group (22 mm in diameter × 2 mm thick) were obtained in stone molds.

Silicone volume and mixing ratios:

For each experimental group, a total volume of 10 cc of silicone was required. Different mixing ratios were used based on the silicone type:

A-2186 specimens used a 10:1 volume ratio of base to catalyst.

A-2000 and A-2006 specimens used a 1:1 volume ratio of base to catalyst.

Preparation for A-2186:

A base mixture of 9 cc was prepared for A-2186 specimens. A total of 0.03 g of each pigment was introduced into the mixture. The pigments included: control group (no pigment); individual pigments: red (R), yellow (Y), and blue (B); and a mixture of 0.01 g each of red, yellow, and blue (R+Y+B). Various thixotropic agents were added in different quantities (0, 1, 2, 3, 4, and 5 drops). Finally, 1 cc of catalyst was added for curing.

Preparation for A-2000 and A-2006:

A total silicone volume of 10 cc was used for A-2000 and A-2006. This consisted of a mixture of 5 cc of Part A and 5 cc of Part B. Each combination of silicone base, catalyst, pigments, and thixotropic agents underwent manual mixing. Mixing was conducted manually using a spatula on a glass slab. The mixing process continued until an even distribution of pigments and thixotropic agents was achieved, resulting in uniform color dispersion within the silicone mixture.

**Figure 1:**
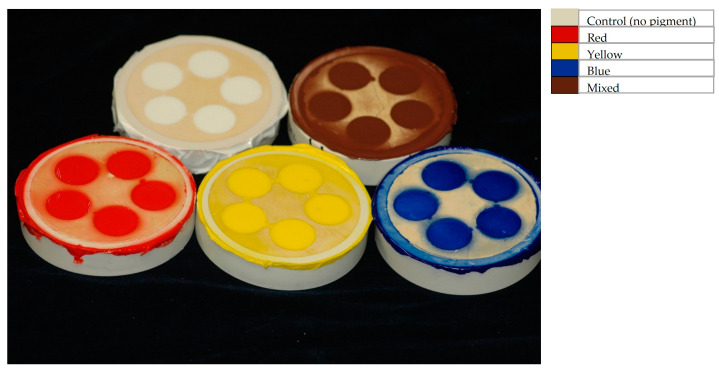
Examples of pigmented silicone specimens in situ within the molds.

The mixtures were loaded into a plastic syringe (Sherwood Medical Co Inc, Saint Louis, MO, USA) and injected into each gypsum mold. Then, the silicone molds were loaded into flasks, then the flasks were tightened in a denture flask press. The material was allowed to polymerize at room temperature for 24 h. After 24 h, the press was placed in a dry hot oven and set at 80 °C for 1 h. All specimens were then trimmed and marked with small notches to classify the number and group (Figure 1).

All specimens were placed in an aging chamber (Ci35 Weather-Ometer; Atlas Electronics, Chicago, IL, USA). Spectrophotometer readings (ColorEye 7000; Macbeth, Newburgh, NY, USA) and CIE L*a*b* color values were measured following the artificial aging as previously described [28]. The position of the specimens was the same for each data-recording interval. The values of L*, a*, and b* were entered into a spreadsheet program (Microsoft Excel, Microsoft Office 2000 Professional; Microsoft Corp, Redmond, Washington) for the calculation of the color change (ΔE*) using the following formula: ΔE* = [(ΔL*)2 + (Δa*)2 + (Δb*)2]1/2, where ΔL* is the change in L* between the interval of interest and baseline, Δa* is the change in a* between the interval of interest and baseline, and Δb* is the change in b* between the interval of interest and baseline.

The statistical analysis was performed by using the R Statistical Software program (R Core Team, 2020) (R Foundation for Statistical Computing, Vienna, Austria) [29]. Four-way ANOVA and Fisher’s PLSD tests were performed to determine if there were statistically significant differences (*p* < 0.05) among the silicone types, the amount of thixotropic agent used, and coloring pigments at each irradiance energy level. Four-way interactions (silicone, aging, pigment, and drop) were performed by using Type II tests.

## 3. Results

The 50:50% perceptibility (ΔE* = 1.1) and acceptability thresholds (ΔE* = 3.0) were used for the interpretation of the data of color changes in this study. The three different maxillofacial silicone elastomers examined displayed significantly varied color changes at irradiance levels of 150, 300, and 450 kj/m^2^ (Table 1, Table 2 and Table 3). Surprisingly, A-2006 exhibited unexpected results in terms of the color stability when the thixotropic agent was added at different concentrations. In particularly, the A-2006 silicone elastomer demonstrated significant color differences, where increasing the quantity of thixotropic agent from 2 to 3 drops, or from 4 to 5 drops, resulted in the color change for the mixed-pigmentation groups to reduce in A-2006 at all irradiance energy levels. Conversely, there was an increase in color change for A-2000 and A-2186 with the addition of thixotropic agent, ranging from 0 to 5 drops at each irradiance level for mixed pigmentations.

Table 1 shows the color change (ΔE*) mean values and standard deviations of the materials at a 150 kj/m^2^ irradiance level. According to this result, only the yellow-pigmented A-2000 specimens with 4 drops of thixotropic agent exceeded (ΔE* = 3.37) the acceptability threshold. However, all the specimens within each group demonstrated acceptable color changes at the 150 kj/m^2^ irradiance level. Furthermore, among the silicone elastomers tested, A-2186 was the only one that stayed below the perceptibility threshold of ΔE* = 1.1, even with the addition of 3 and 4 drops of thixotropic agent.

Table 2 presents the ΔE* mean values and standard deviations of the materials at the 300 kj/m^2^ irradiance level. It is evident that adding up to 3 drops of thixotropic agent results in acceptable color changes for all silicones, with values below 3 ΔE*. However, when 4 drops of thixotropic agent were added, severe color changes were observed in the red, yellow, and blue pigments of the A-2000 silicone, as well as the red pigment of the A-2006 silicone. Furthermore, the addition of 5 drops of thixotropic agent led to values exceeding 3 ΔE* in the yellow A-2000 and mixed-pigment A-2186 silicones. The other subgroups showed acceptable color changes at a 300 kj/m^2^ irradiance level.

Table 3 displays the color stability of the materials at the 450 kj/m^2^ irradiance level. The addition of 0–3 drops of thixotropic agent revealed an acceptable color change in all subgroups for each silicone. The addition of 0–3 drops of thixotropic agent resulted in acceptable color changes in all subgroups for each silicone. However, the addition of 4 drops of thixotropic agent led to color changes exceeding the acceptable threshold in the yellow pigment of A-2000, the red pigment of A-2006, and both the yellow and red pigments of A-2186 silicone. Furthermore, when 5 drops of thixotropic agent were added, color changes exceeding the acceptability threshold were observed in the yellow and 5-drops red pigmented A-2000, as well as in the yellow-, red-, and blue-pigmented A-2186 silicone.

Table 4 presents the results of a comprehensive analysis of the 4-way interactions among the groups. The statistical analysis demonstrates the presence of significant 4-way interactions among the explanatory variables.

**Figure 2:**
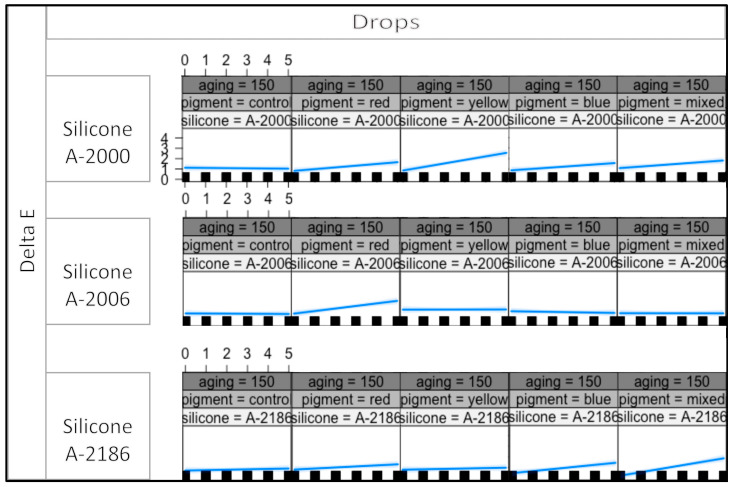
The plot for the 150 kj/m^2^ irradiance level, showing the color change for each pigment group and all silicones.

**Figure 3:**
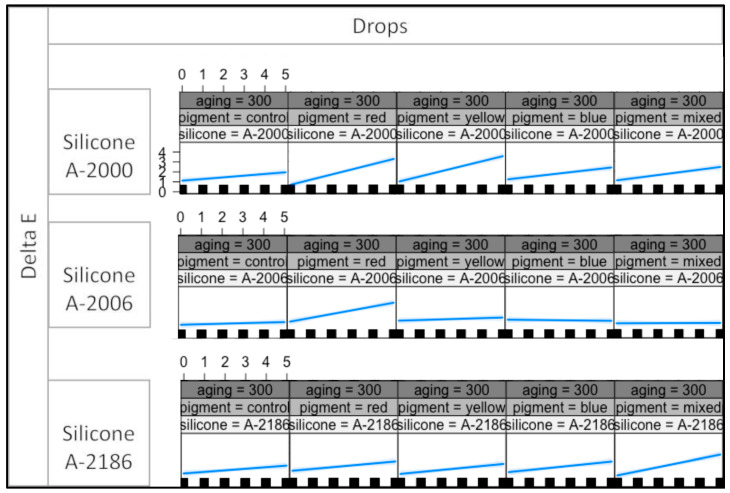
The plot for the 300 kj/m^2^ irradiance level, showing the color change for each pigment group and all silicones.

**Figure 4:**
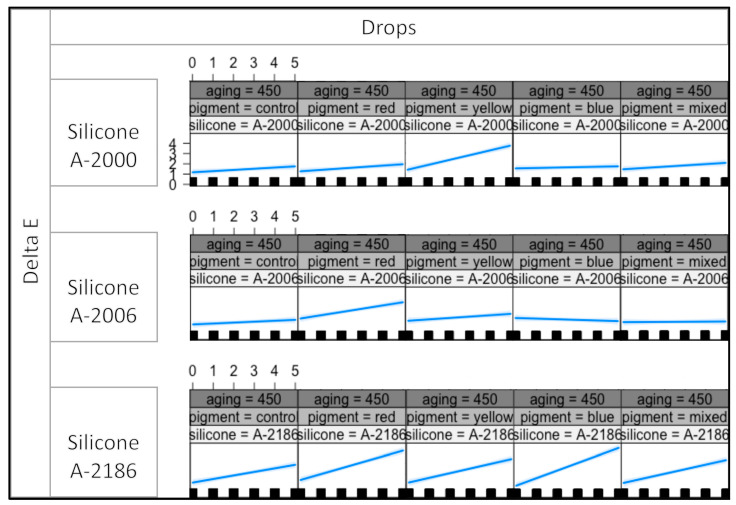
The plot for the 450 kj/m^2^ irradiance level, showing the color change for each pigment group and all silicones.

This indicates that the effects on the response variables are contingent upon specific combinations of all four variables. Figure 2, Figure 3 and Figure 4 presents the interaction plot resulting from the statistical analysis conducted in this study. The plotted data effectively illustrate the varying outcomes for the response variables based on different combinations of the four explanatory variables.

The plot effectively illustrates the impact of the thixotropic agent’s quantity in A-2000, A-2006, and A-2186 silicones on the response variable. It becomes evident that varying the amount of thixotropic agent leads to distinct effects on the outcome. In contrast, the type of pigment exhibited a minimal or negligible influence on the color stability. The interaction plot indicates that the color stability is primarily governed by the silicone used rather than the pigment type. Notably, color stability was found to be dependent on the specific silicone. At radiation levels of 300 and 450 kj/m^2^, A-2186 silicone demonstrated the highest degree of color change among all the pigment groups. Moreover, the plot exhibits an upward trend as the drop number increases, indicating a positive correlation between the drop number and color change. These observations highlight the critical role of thixotropic agent quantity and silicone type in determining color stability, while pigment type has limited significance in this context. The findings underscore the need for the careful consideration of these factors in practical applications, and particularly emphasize the importance of selecting an appropriate silicone type to achieve the desired color stability in the given conditions.

Figure 2 shows the plot for the 150 kj/m^2^ irradiance level. It is obvious that the plot curves are mostly similar to each other in the same row, and in the same column. It can be concluded that the thixotropic agent’s quantity plays a significant role in the color change for each silicone for each pigment type at the 150 kj/m^2^ irradiance level.

According to Figure 2, Figure 3 and Figure 4, the color change was more dependent on the thixotropic agent quantity in the red pigmentation group for A-2006 at all three irradiance levels (150, 300, and 450 kj/m^2^). A noteworthy color change was observed in the red pigmentation group for A-2006 at all three irradiance levels (150, 300, and 450 kj/m^2^). A similar effect was observed in the yellow pigmentation group for the A-2000 silicone. These findings suggest that these specific pigments (yellow and red) seem to have an impact on the color stability levels of A-2000 and A-2006, respectively, in conjunction with the quantity of thixotropic agent added.

The most dramatic color changes occurred in the yellow pigmentation group for A-2000, which further increased with the addition of thixotropic agent, at each energy level. When it came to the A-2006 silicone, the red pigmentation group demonstrated the least color stability at all energy levels. Additionally, these color changes showed a gradual increment as the aging process progressed from 150 to 450 kj/m^2^ irradiance levels. Interestingly, the color change did not increase with the addition of thixotropic agent to each pigmentation group, except for the red pigment in the A-2006 silicone. This outcome was consistent across different irradiance levels for A-2006. These findings suggest that the red pigment has a strong interaction with the thixotropic agent, resulting in a noticeable effect on the color stability, as the amount of thixotropic agent or the irradiance level does not significantly affect the color change.

## 4. Discussion

The primary challenge with facial prostheses is the need for replacement, often due to the esthetic loss caused by discoloration. In clinical practice, the crucial factor influencing the patient’s acceptance of a prosthesis is its color stability [19,30]. In the current study, the impact of various hours of aging and thixotropic agents on the color stability of three different maxillofacial silicone elastomers was assessed. Regardless of whether the specimens were pigmented or not, all of them showed different color stability levels, regardless of whether these changes occurred within the perceptible threshold. The hypothesis of this study was rejected as different amounts of thixotropic agent had varying effects on the color change of the silicone elastomers.

The presence of a significant four-way statistical interaction indicates that the response variables are intricately linked and contingent upon specific levels and combinations of all four variables. In other words, the outcomes observed in the response variables are highly influenced by the interplay between these four factors [31]. As a result, the interaction plots that are presented in Figure 2, Figure 3 and Figure 4 assume critical importance in this study. It serves as a visual tool to elucidate the diverse combinations of the four variables that result in changes in the response variable. A visual representation helps to discern the influential factors and their respective contributions to the observed outcomes. Statistical interactions are widely recognized and expected in complex systems, confirming the presence of the interaction between the groups on three levels. However, despite the significant interactions, the specific sources of differences observed in the interaction table remain indeterminate. In conclusion, the findings emphasize the importance of considering multi-variable interactions in our analysis, and the plot aids in understanding the nuanced relationships among the variables. Nonetheless, further investigations are warranted to pinpoint the exact origins of the observed differences, which can provide valuable insights for future research.

The plot effectively showcased how the response variable changed in response to different levels of the four variables, providing valuable insights into the nature and magnitude of their combined effects. This visual representation enhanced the interpretability and understanding of the intricate relationships among the variables, which may have not been as apparent from the numerical data alone. Hence, the utilization of the interaction plot in this study is scientifically justified and essential for comprehending the complex interdependencies between the four variables and their joint influence on the response variable. Therefore, the plot interaction plots, perceptibility and acceptability thresholds, as well as statistical differences were used for the interpretation of the results in this study.

Various studies have reported perceptible color changes in maxillofacial silicone prostheses, with values ranging from ΔE* 1 to 3 units [14,28]. The differences were likely due to the use of different pigments and silicone materials. Paravina et al. [25] found that color changes of 1.1 units were perceptible and 3 units were acceptable for light skin-colored maxillofacial silicones. In the present study, the addition of more than 2 drops of thixotropic agent resulted in a significant color change in the A-2186 silicone for the mixed-pigmentation group at the 450 kj/m^2^ irradiance level. Specifically, when the thixotropic agent quantity was increased to 5 drops, the mixed-pigmented A-2186 silicone exhibited considerable color changes (ΔE* = 3.53 ± 0.4 and 3.54 ± 0.33) at 300 and 450 kj/m^2^, respectively, surpassing the acceptability threshold.

Silicone elastomers are commonly pigmented both internally and externally to match the surrounding tissues when used in prostheses. However, over time, this initial pigmentation can undergo color degradation due to various factors, such as UV-light exposure, moisture, temperature changes, pollutants from the environment, and microscopic residues from the skin, as well as the use of cleaning and disinfecting agents [21]. Among these factors, exposure to light energy has the most significant impact on color changes in high-temperature vulcanized (HTV) and room-temperature vulcanized (RTV) silicones [31,32]. In our study, we measured the aging of specimens using kj/m^2^ instead of counting the total hours in an aging chamber. This method allowed for better comparisons of dental materials’ color stability [22]. It is essential to exercise caution when interpreting the results obtained through various testing methods, as they may not perfectly represent real-time conditions [22]. To simulate different outdoor conditions, we employed various levels of irradiance energy (150, 300, and 450 kj/m^2^). The color degradation in all groups increased as the irradiance level increased from 150 to 450 kj/m^2^ as expected, which indicated that aging had a strong effect on the color stability of the materials, regardless of the pigment incorporation. It is noteworthy that irradiation at an intensity of 450 kj/m^2^ resulted in color changes not only in the pigmented silicone groups, but also in the control groups of the A-2186 silicone elastomer with the addition of thixotropic agent. The influence of thixotropic agent quantity was more pronounced in the control group at 450 kj/m^2^, compared to the group at the 150 kj/m^2^ irradiance level for the A-2186 silicone. Conversely, this observation did not hold true for A-2000 or A-2006 silicones, as the control specimens showed no statistically significant color changes with varying thixotropic agent drop quantities at the 450 kj/m^2^ irradiance level. In this regard, the higher level of light energy could break the chain bonds of the silicone, leading to the elastomer’s decomposition, which, in turn, may result in color degradation. In this context, the increased intensity of light energy has the potential to disrupt the chain bonds within the silicone structure. This disruption can initiate the decomposition of the elastomer, subsequently contributing to color degradation. Notably, distinctions in cross-linking and polymerization degrees may be contingent on the material’s chemical composition and mixing ratios. These variations in material characteristics may elucidate the observed discrepancies in the color degradation among A-2000, A-2006, and A-2186 silicone types. Furthermore, to comprehensively understand the intricate composition effects of these materials, future studies within the realm of materials science are recommended. Such investigations can delve into the precise interplay between the material’s constituents, their proportions, and their chemical interactions. This avenue of exploration would enable a more nuanced comprehension of the underlying mechanisms driving color degradation and provide valuable insights for enhancing the stability of silicone elastomers under similar conditions.

The thixotropic agent is formulated to swiftly provide the silicone elastomer with a smooth and creamy texture upon incorporation, effectively increasing its viscosity during molding [27,33,34]. Additionally, it facilitates the creation of adequate pressure within the mold, reducing the presence of air bubbles. This agent can serve as an alternative to vacuum deairing. The recommended dosage is one to two drops per 10 g of silicone. According to a survey conducted by Cardoso et al. [27], 58% of clinicians used a thixotropic agent, with 54% opting for one to two drops per 10 g of silicone, while 4% reported using more than two drops per 10 g.

Thixotropic agent is a silicone liquid that contains dimethyl siloxane, ethoxylated 3 hydroxypropyl-terminated, polyethylene oxide mono alkyl ether, and polyethylene glycol, which can contain high quantities of hydroxyl ions [26]. These hydroxyl ions may interact with Si-O bonds and cause an increase in the viscosity of the uncured material. A-2000, A-2186, and A-2006 are platinum-based room-temperature vulcanized (RTV) maxillofacial silicone elastomers, commonly used in combination with thixotropic agents for making maxillofacial prostheses. The specific chemical composition and processing of these silicone elastomers play a crucial role in determining their physical and mechanical properties. In this study, silicone elastomer specimens were polymerized in stone molds to replicate clinical practice, and they were exposed to varying amounts of light energy. It is well known that silicone does not fully polymerize in stone molds, and polymerization continues even after deflasking [35,36]. During this ongoing polymerization, subproducts may be released, and there is a possibility that these interact with the thixotropic agent, causing alterations in the chromatic patterns of the silicone elastomers. Furthermore, Bibars et al. [33] mixed 0.06 g of a thixotropic agent with 30 g of M511, A-2000, and Z004 (including 0.12 g of Technovent P402 silicone pigment) and observed that the thixotropic agent reduced the tear strength and percent elongation of all the tested silicones. Consequently, it is essential for clinicians to exercise extra caution when adding thixotropic agents, as they can have adverse effects on the properties of commonly used silicones, particularly A-2000, M511, Z004, and VST50. This result is consistent with the results obtained in this study. In the current study, the quantity of thixotropic agent (thixo) had varying effects on the three different silicones, suggesting a strong chemical interaction between the thixotropic agent and the silicone matrix. These findings underscore the importance of understanding and considering the specific chemical interactions when using thixotropic agents with different silicone materials in maxillofacial prosthetic applications.

The color of an object, as perceived by the human eye, is determined by the wavelength of light reflected from its surface. White objects reflect all light, while objects of other colors absorb certain wavelengths. This absorbed light carries energy that can impact the physical properties of the object, leading to color fading [4]. The higher the wavelength, the greater the energy absorbed by the object [32]. Consequently, red objects would be expected to experience the highest degree of color degradation, while blue objects would be affected the least, considering the light energy they absorb. Similarly, in our study, A-2006 silicone exhibited the most substantial color changes in all the pigmentation groups except for the red pigmentation group, in accordance with the increasing amount of thixotropic agent. In addition, the same thing occurred in the yellow pigmentation groups for A-2000, leading to the most color change as compared to the control group at each irradiance level. Interestingly, when the thixotropic agent was added to the silicone and pigment mixtures, the fading process of the color intensified even further in the A-2000 and A-2186 silicones. This observation might be attributed to the chemical interactions between the silicone, pigments, and thixotropic agent. Because different silicones have distinct matrix structures [8,33,34,37,38,39], the physical properties can be different for each silicone. Different chemical bonds can be responsible for various color stability results in each silicone group.

Aging at different irradiance levels resulted in varying outcomes concerning the color changes for each silicone. At radiation levels of 300 and 450 kj/m^2^, A-2186 silicone showed the most significant color change in all pigment groups. This suggests that aging at higher energy levels leads to more pronounced physical degradation in A-2186, especially when combined with an increased amount of thixotropic agent in all pigmentation groups. Furthermore, as shown in Figure 2, Figure 3 and Figure 4, A-2006 exhibits a consistent color change at each irradiance energy level, indicating that A-2006 can be the most color-stable silicone elastomer based on the findings of this study. These results emphasize the significance of considering the specific silicone materials and their interactions with pigments and thixotropic agents when evaluating color stability in maxillofacial prosthetic applications. Furthermore, it is important to note that artificial aging serves as a controlled method but may not entirely replicate natural weathering conditions. In order to comprehensively understand exposure times and their correspondence to natural weathering, further research is needed. We can simulate natural weathering by conducting artificial aging at various exposure parameters; however, achieving the direct simulation of natural weathering is complex due to the multitude of atmospheric variables. At present, there is no consensus on whether materials act differently under artificial aging or outdoor weathering conditions. To date, there is only one report evaluating the effects of both artificial aging and outdoor weathering on the color stability of different maxillofacial elastomers [28]. Different color changes may be obtained from outdoor weathering or artificial aging because of the different response outcomes of the materials or the aging conditions. On the other hand, to the best of authors’ knowledge, there is no report performing a comparative analysis of the different materials and pigmentation when exposed to both artificial aging and outdoor weathering.

Intrinsic pigments can affect the mechanical or physical properties of silicone elastomers [40,41]. In certain cases, different pigments can offer color protection in hot and humid climates [42,43]. The effect of pigments on the color change of silicone elastomers has been reported in previous studies [13,22,32,40,44,45,46,47,48]. The degree of polymerization and physical properties of the polymer can be directly affected by the pigments, induced by progressive cross-linking within the matrix [49]. Determining the precise composition of various compounds and products used in a silicone matrix poses a challenge. In silicone elastomers, different types of pigments are incorporated, altering the composition of paints to achieve enhanced quality and stability [50]. Thus, the custom analysis of these paints becomes crucial for conservation purposes, ensuring the verification of any changes in their composition within the silicone matrix. In this study, for the red-pigmented A-2006 silicone, there was a significant increase in the color stability as the thixotropic agent amount increased from 4 to 5 drops, at both the 300 and 450 irradiance levels. This suggests that the thixotropic agent amount should either be 0–3 or 5 drops when using red pigments in A-2006 silicone, as 4 drops could have a detrimental effect on the silicone’s color stability. Additionally, it can be inferred that the high amount of red pigment is critical when using A-2006 silicone. On the other hand, when an increased amount of yellow pigment is used in the A-2000 silicone, the thixotropic agent amount should not exceed 3 drops to maintain acceptable color stability. Indeed, these findings emphasize the critical importance of carefully considering the specific combination of pigments and silicone materials, considering their interactions, as well as the influence of the thixotropic agent. Such considerations are essential to ensure optimal color stability in the fabrication of maxillofacial prostheses. By understanding and managing these factors, clinicians can enhance the longevity and aesthetic appeal of facial prostheses, ultimately improving patient satisfaction and acceptance.

The combination of different products can alter chemical compositions and either enhance or compromise overall material properties. Recent studies have demonstrated that many clinicians have routinely used this agent for several years in their practice, often in combination with other silicone elastomers. However, our study highlighted that the thixotropic agent could significantly impact the color stability of silicone elastomers in different ways. Special consideration should be given to the combination of yellow pigment with A-2000 silicone and red pigment with A-2006 silicone, as these combinations demonstrated dependency on specific pigment incorporations for the color stability in this study. Therefore, clinicians should exercise caution and seek information on the potential effects of modifying agents before incorporating new products into their practices. Being aware and well-informed about such interactions is crucial to maintaining the desired material performance in clinical applications.

## 5. Conclusions

Based on the findings of this study, the following conclusions can be drawn:

1. The amount of thixotropic agent plays a crucial role in determining the color stability of different silicone elastomers pigmented with various intrinsic pigments. The thixotropic agent amount has a more significant impact on the color stability than the type of pigment used in the silicone elastomers.
2. In the mixed-pigmentation group, the addition of 4 drops of thixotropic agent resulted in color changes exceeding 3ΔE* only in the A-2186 silicone at the 300 and 450 kj/m^2^ energy levels. However, the color stability of mixed-pigmented A-2000 and A-2006 remained within the acceptable threshold at all irradiance levels in this study.
3. At the 150 kj/m^2^ energy level, the addition of 0–5 drops of thixotropic agent did not significantly affect the color change in most of the silicone and pigment groups. All silicone and pigment groups, except for the yellow-pigmented A-2000, exhibited acceptable color changes at the 150 kj/m^2^ level.
4. Yellow-pigmented A-2000 silicone with 4 drops of thixotropic agent showed color changes above the acceptability threshold at all three irradiance levels in this study.
5. Among all the silicones, A-2006 exhibited the highest color stability with an increasing amount of thixotropic agent at each energy level. Conversely, A-2186 was more susceptible to the effects of an increased amount of thixotropic agent in each pigmentation group.
6. The addition of thixotropic agents had a more adverse impact on A-2186 silicone elastomers compared to the other elastomers tested in this study at 300 and 450 kj/m^2^ energy levels.
7. Clinicians should take caution when selecting the combination of silicone, thixotropic agent amount, and specific pigments during the fabrication of maxillofacial silicone prostheses. These factors can significantly influence the color stability of the prosthesis and should be carefully considered to achieve optimal results.

## Figures and Tables

**Table 1:** Means and SDs for all pigmentation groups in each thixotropic agent group for each silicone for the 150 kj/m^2^ aging process.

Silicone	Pigments	Control Thixo	1-Drop Thixo	2-Drop Thixo	3-Drop Thixo	4-Drop Thixo	5-Drop Thixo
A-2000	Control	0.98 (0.25)	1.15 (0.41)	0.98 (0.16)	1.47 (0.64)	1.01 (0.61)	0.83 (0.24)
A-2000	Red	0.98 (0.19)	0.83 (0.37)	1.05 (0.17)	1.05 (0.18)	2.07 (0.32)	1.41 (0.10)
A-2000	Yellow	1.08 (0.32)	0.90 (0.19)	1.19 (0.16)	1.78 (0.31)	3.37 (0.31)	1.88 (0.37)
A-2000	Blue	0.74 (0.55)	0.78 (0.27)	1.16 (0.23)	1.78 (0.50)	1.84 (0.68)	0.97 (0.11)
A-2000	Mixed	1.14 (0.30)	0.73 (0.30)	1.43 (0.70)	1.96 (0.64)	2.24 (0.28)	1.15 (0.15)
A-2006	Control	0.83 (0.21)	0.93 (0.14)	1.18 (0.21)	0.84 (0.28)	0.96 (0.29)	0.81 (0.44)
A-2006	Red	0.91 (0.11)	1.32 (0.33)	1.42 (0.42)	1.34 (0.35)	2.05 (0.60)	2.18 (0.16)
A-2006	Yellow	0.99 (0.40)	1.75 (0.18)	1.35 (0.44)	1.17 (0.52)	1.43 (0.44)	1.24 (0.28)
A-2006	Blue	1.35 (0.46)	0.89 (0.37)	1.34 (0.24)	0.89 (0.52)	0.61 (0.19)	1.38 (0.33)
A-2006	Mixed	0.92 (0.56)	0.96 (0.24)	1.22 (0.19)	0.66 (0.06)	1.13 (0.37)	0.91 (0.15)
A-2186	Control	0.83 (0.08)	0.68 (0.27)	0.76 (0.12)	0.64 (0.13)	0.97 (0.02)	0.94 (0.06)
A-2186	Red	0.76 (0.21)	0.90 (0.09)	1.25 (0.58)	0.94 (0.07)	0.99 (0.07)	1.45 (0.17)
A-2186	Yellow	0.97 (0.04)	0.72 (0.13)	0.72 (0.12)	0.86 (0.08)	0.91 (0.16)	1.09 (0.16)
A-2186	Blue	0.57 (0.22)	0.74 (0.15)	0.73 (0.09)	0.93 (0.13)	0.91 (0.06)	1.79 (0.56)
A-2186	Mixed	0.44 (0.18)	0.55 (0.24)	0.79 (0.13)	0.95 (0.04)	1.14 (0.25)	2.36 (0.47)

**Table 2:** Means and SDs for all pigmentation groups in each thixotropic agent group for each silicone for the 300 kj/m^2^ aging process.

Silicone	Pigments	Control Thixo	1-Drop Thixo	2-Drop Thixo	3-Drop Thixo	4-Drop Thixo	5-Drop Thixo
A-2000	Control	1.11 (0.21)	1.05 (0.31)	1.51 (0.29)	1.90 (0.37)	1.58 (1.00)	1.89 (0.62)
A-2000	Red	0.76 (0.09)	0.95 (0.24)	1.47 (0.25)	2.39 (0.38)	3.30 (0.65)	2.80 (0.48)
A-2000	Yellow	1.38 (0.26)	1.19 (0.31)	1.55 (0.28)	2.23 (0.31)	4.12 (0.34)	3.04 (0.41)
A-2000	Blue	1.05 (0.30)	1.32 (0.23)	1.46 (0.36)	2.31 (0.29)	3.09 (0.67)	1.49 (0.16)
A-2000	Mixed	1.39 (0.31)	0.69 (0.44)	1.48 (0.23)	2.38 (0.44)	2.78 (0.35)	1.84 (0.67)
A-2006	Control	1.06 (0.14)	1.14 (0.31)	1.62 (0.24)	1.15 (0.40)	1.25 (0.14)	1.45 (0.56)
A-2006	Red	1.32 (0.50)	1.65 (0.23)	2.46 (0.23)	2.08 (0.24)	4.22 (0.16)	2.49 (0.40)
A-2006	Yellow	1.14 (0.28)	2.33 (0.28)	1.53 (0.32)	1.52 (0.50)	1.45 (0.16)	2.11 (0.34)
A-2006	Blue	1.42 (0.26)	1.95 (0.31)	1.82 (0.20)	1.42 (0.23)	0.73 (0.12)	2.08 (0.23)
A-2006	Mixed	1.21 (0.67)	1.16 (0.26)	1.45 (0.26)	1.02 (0.11)	1.70 (0.32)	1.01 (0.13)
A-2186	Control	1.36 (0.12)	1.13 (0.21)	1.62 (0.23)	0.88 (0.15)	1.66 (0.10)	2.24 (0.11)
A-2186	Red	1.58 (0.36)	1.51 (0.12)	1.57 (0.18)	1.51 (0.21)	1.97 (0.17)	2.56 (0.16)
A-2186	Yellow	1.28 (0.10)	1.10 (0.10)	1.23 (0.17)	1.40 (0.15)	2.10 (0.26)	1.99 (0.17)
A-2186	Blue	1.32 (0.21)	1.642 (0.25)	1.33 (0.15)	1.49 (0.19)	2.24 (0.29)	2.35 (0.87)
A-2186	Mixed	1.00 (0.07)	1.57 (0.02)	1.54 (0.19)	1.70 (0.20)	1.95 (0.11)	3.53 (0.40)

**Table 3:** Means and SDs for all pigmentation groups in each thixotropic agent group for each silicone for the 450 kj/m^2^ aging process.

Silicone	Pigments	Control Thixo	1-Drop Thixo	2-Drop Thixo	3-Drop Thixo	4-Drop Thixo	5-Drop Thixo
A-2000	Control	1.03 (0.13)	1.22 (0.35)	1.75 (0.57)	1.46 (0.62)	1.17 (0.90)	1.91 (0.50)
A-2000	Red	1.49 (0.11)	1.18 (0.35)	1.27 (0.23)	1.29 (0.27)	2.680 (0.59)	1.55 (0.20)
A-2000	Yellow	1.35 (0.16)	1.67 (0.14)	2.56 (0.50)	2.66 (0.39)	3.66 (0.09)	3.41 (0.62)
A-2000	Blue	1.73 (0.46)	1.01 (0.44)	1.46 (0.36)	2.25 (0.37)	1.89 (0.40)	1.33 (0.21)
A-2000	Mixed	1.65 (0.11)	0.85 (0.27)	1.92 (0.21)	1.78 (0.48)	2.89 (1.07)	1.33 (0.07)
A-2006	Control	0.98 (0.17)	1.44 (0.24)	1.64 (0.07)	1.78 (0.67)	1.61 (0.03)	1.48 (0.49)
A-2006	Red	1.60 (0.25)	2.25 (0.07)	2.42 (0.31)	2.63 (0.82)	4.42 (0.18)	2.47 (0.35)
A-2006	Yellow	1.58 (0.19)	2.15 (0.21)	1.63 (0.24)	1.99 (0.46)	1.92 (0.25)	2.59 (0.34)
A-2006	Blue	1.94 (0.48)	2.37 (0.24)	1.79 (0.25)	0.87 (0.25)	1.14 (0.28)	2.44 (0.33)
A-2006	Mixed	1.68 (0.66)	1.50 (0.24)	1.48 (0.20)	1.10 (0.09)	1.92 (0.26)	1.59 (0.38)
A-2186	Control	1.39 (0.21)	1.68 (0.16)	1.83 (0.19)	1.42 (0.30)	2.84 (0.25)	3.13 (0.06)
A-2186	Red	2.04 (0.13)	1.89 (0.10)	2.18 (0.21)	2.43 (0.22)	3.75 (0.27)	4.76 (0.26)
A-2186	Yellow	1.65 (0.21)	1.38 (0.09)	1.95 (0.08)	2.00 (0.22)	3.30 (0.23)	3.58 (0.26)
A-2186	Blue	1.66 (0.14)	1.93 (0.23)	1.66 (0.12)	2.01 (0.29)	2.93 (0.23)	5.98 (0.79)
A-2186	Mixed	1.31 (0.35)	1.66 (0.16)	1.90 (0.16)	2.23 (0.20)	2.84 (0.25)	3.54 (0.33)

**Table 4:** Analysis of deviance table (Type II tests) indicating 4-way interactions.

	Sum Sq	Df	F Value	Pr (>F)
Silicone	3.754	2	16.2243	1.142 × 10^−7^
Pigment	50.915	4	110.0260	<2.2 × 10^−16^
Aging	180.545	2	780.3033	<2.2 × 10^−16^
Drops	167.066	5	288.8196	<2.2 × 10^−16^
Silicone:pigment	49.194	8	53.1532	<2.2 × 10^−16^
Silicone:aging	48.259	4	104.2860	<2.2 × 10^−16^
Pigment:aging	6.254	8	6.7571	1.131 × 10^−8^
Silicone:drops	93.160	10	80.5258	<2.2 × 10^−16^
Pigment:drops	38.776	20	16.7586	<2.2 × 10^−16^
Aging:drops	19.605	10	16.9461	<2.2 × 10^−16^
Silicone:pigment:aging	9.601	16	5.1869	1.369 × 10^−10^
Silicone:pigment:drops	81.861	40	17.6898	<2.2 × 10^−16^
Silicone:aging:drops	32.102	20	13.8741	<2.2 × 10^−16^
Pigment:aging:drops	20.504	40	4.4308	<2.2 × 10^−16^
Silicone:pigment:aging:drops	31.827	80	3.4389	<2.2 × 10^−16^
Residuals	124.944	1080		
---				

## Data Availability

The data presented in this study can be obtained from the corresponding author.
